# The impact of fasting on resting state brain networks in mice

**DOI:** 10.1038/s41598-019-39851-6

**Published:** 2019-02-27

**Authors:** Tomokazu Tsurugizawa, Boucif Djemai, Andrew Zalesky

**Affiliations:** 1 NeuroSpin, Commissariat à l’Energie Atomique et aux Energies Alternatives, CEA Saclay, Gif-sur-Yvette, 91191 France; 2 Melbourne Neuropsychiatry Centre and Department of Biomedical Engineering, The University of Melbourne, Victoria, 3010 Australia

## Abstract

Fasting is known to influence learning and memory in mice and alter the neural networks that subserve these cognitive functions. We used high-resolution functional MRI to study the impact of fasting on resting-state functional connectivity in mice following 12 h of fasting. The cortex and subcortex were parcellated into 52 subregions and functional connectivity was measured between each pair of subregions in groups of fasted and non-fasted mice. Functional connectivity was globally increased in the fasted group compared to the non-fasted group, with the most significant increases evident between the hippocampus (bilateral), retrosplenial cortex (left), visual cortex (left) and auditory cortex (left). Functional brain networks in the non-fasted group comprised five segregated modules of strongly interconnected subregions, whereas the fasted group comprised only three modules. The amplitude of low frequency fluctuations (ALFF) was decreased in the ventromedial hypothalamus in the fasted group. Correlation in gamma oscillations derived from local field potentials was increased between the left visual and retrosplenial cortices in the fasted group and the power of gamma oscillations was reduced in the ventromedial hypothalamus. These results indicate that fasting induces profound changes in functional connectivity, most likely resulting from altered coupling of neuronal gamma oscillations.

## Introduction

Physiological states such as satiety and hunger can strongly influence goal-directed behavior, including learning, memory and cognitive performance^1–3^. The influence of these physiological states on cognition is partially mediated by an acute decrease in glucose levels and alterations in hormones transported by the blood^4,5^. Given that resting-state functional connectivity is associated with cognition^6^, we hypothesized that fasting can induce transient reorganization of functional brain connectivity, and thus influence cognitive performance.

We use high-field functional MRI (fMRI)^7,8^ to measure low-frequency synchronization (0.01–0.1 Hz) in intrinsic neural activity between pairs of gray matter regions. This synchronization is known as resting-state functional connectivity^9^. Functional connectivity has been widely studied in aging, disease and pharmacological rodent models as well as humans^7,8,10–15^. However, the impact of fasting on functional brain networks more broadly has not been studied in rodents. In contrast, human fMRI studies report that overnight fasting is associated with significant increases in functional connectivity between several regions, particularly the superior frontal gyrus and insular cortex^16^. The ingestion of glucose leads to decreased activity and connectivity in the networks linked to satiation^17^. Collectively, these fMRI studies indicate that fasting is associated with regionally circumscribed increases in functional connectivity, but it remains unclear whether these changes are neuronal in origin or due to altered neurovascular coupling resulting from decreased blood glucose levels.

In the present study, using high-field fMRI and electrophysiological techniques, we aim to investigate the architecture of functional brain networks in fasted mice and to understand the neuronal/neurovascular mechanisms of fasting-induced changes in functional connectivity. We use the network-based statistic to identify resting-state functional connections that are significantly modulated by fasting and employ network modularity analysis to assess whether fasting impacts brain network modular organization. We then measure local field potential (LFP) to test whether our fMRI findings can be replicated in the absence of neurovascular effects.

## Results

### Blood glucose levels and body weight

**Figure 1:**
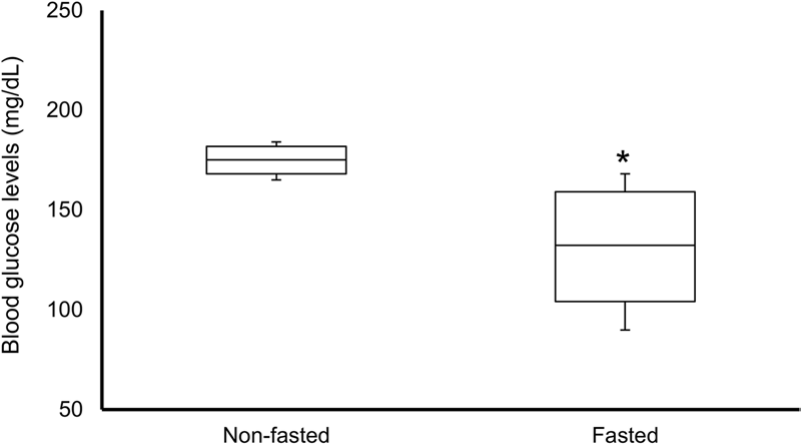
Blood glucose levels in non-fasted (n = 4) and fasted (n = 4) groups. Data are expressed as mean ± SEM. *p < 0.05 by t-test.

Blood glucose levels provide a valid marker of fasting and dynamically vary across the feeding cycle. We therefore measured blood glucose levels in the fasted and non-fasted groups at the time of MRI acquisition (Fig. 1). Blood glucose levels in the non-fasted group were significantly higher than the fasted group (175 ± 5 mg/dL in non-fasted group and 131 ± 19 mg/dL in fasted group; p = 0.03).

### Fasting increases global functional connectivity

**Figure 2:**
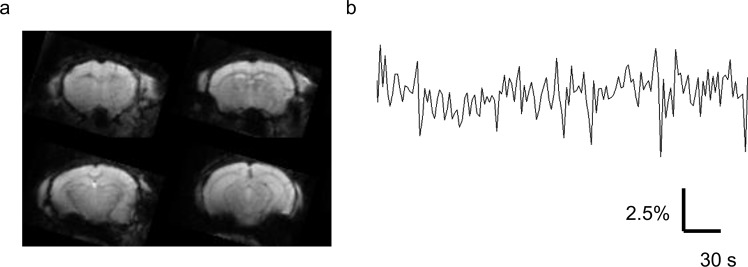
(**a**) Multiple slices of unprocessed functional images of a representative mouse. (**b**) Representative spontaneous fluctuation of BOLD signals in the left retrosplenial cortex. Vertical axis, percent change of BOLD signals.

**Figure 3:**
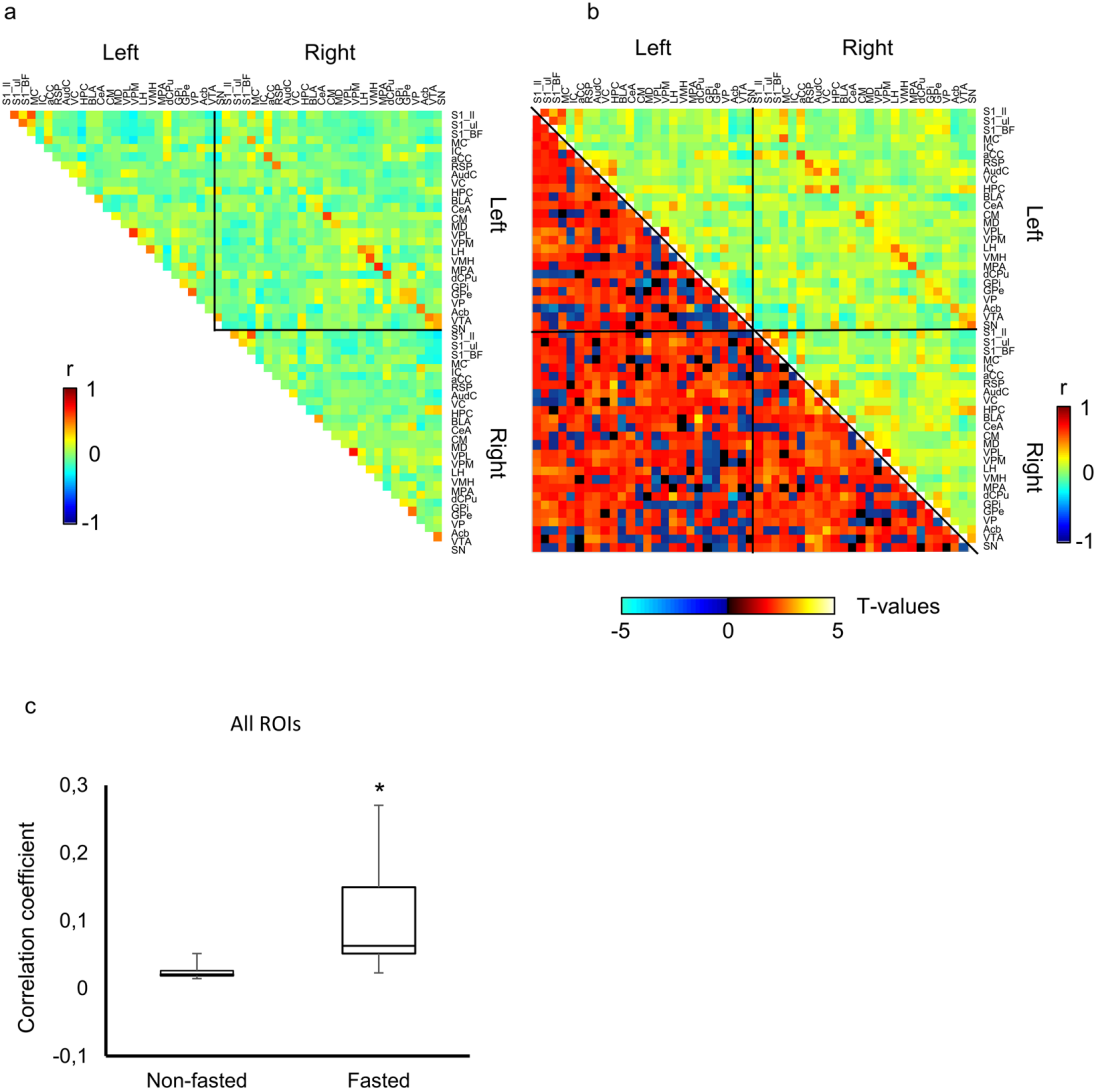
ROI-ROI correlation coefficient matrices in (**a**) non-fasted and (**b** right) fasted groups, and (**b** left) t-statistic values of each ROI-ROI correlation coefficients between fasted and non-fasted groups. Vertical color bar represents the correlation coefficient. Horizontal color bar represents the t-values. Labels of the ROIs are shown in Table 1. (**c**) Averaged correlation coefficients of all networks in fasted and non-fasted groups. Data are expressed as mean ± SEM. *p < 0.05, by t-test.

We investigated whole-brain functional connectivity in fasted and non-fasted mice (Fig. 2a,b). Cortical and subcortical gray matter was parcellated into 52 regions of interest (ROIs) and the Pearson correlation coefficient was computed between all pairs of ROI-averaged time-series to yield a 52 × 52 connectivity matrix (see Methods). A connectivity matrix was computed for each mouse and group-averaged connectivity matrices were then determined (Fig. 3a, non-fasted; 3b, fasted; Data are expressed as mean ± SEM.). The connectivity matrices were ordered such that the first 26 row/columns correspond to left-hemisphere ROIs, while the last 26 rows/columns correspond to right-hemisphere ROIs. The strongly connected off-diagonal pairs of ROIs evident in the connectivity matrices correspond to contralateral homologues. It can be observed that functional connectivity is increased for many pairs of ROIs in the fasted group. Indeed, this was confirmed with rejection of the null hypothesis of equality in the mean functional connectivity averaged across all pairs of ROIs between the fasted and non-fasted groups (Fig. 3c).

### Fasting induces functional connectivity increases between hippocampus, retrosplenial cortex and visual cortex

**Figure 4:**
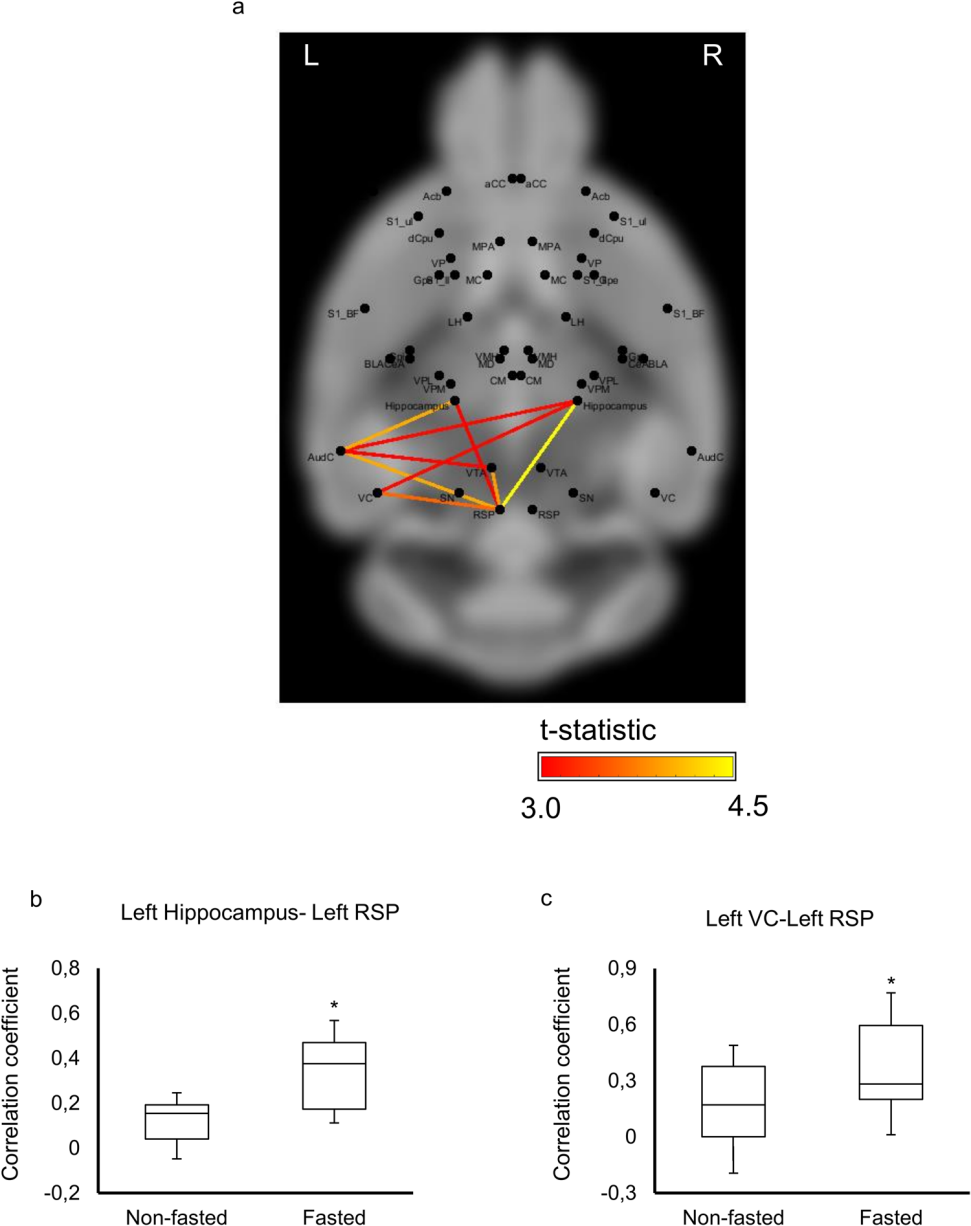
(**a**) Comparison of functional connectivity strength. The averaged correlation coefficients in (**b**) left hippocampus-left retrosplenial cortex (RSP) and (**c**) left visual cortex (VC)-left retrosplenial cortex (RSP) in non-fasted and fasted groups. Data are expressed as mean ± SEM. *p < 0.05 by t-test. Background image from http://imaging.org.au/AMBMC/Model.

Having found whole-brain increases in functional connectivity induced by fasting, we next sought to test whether these global changes could be localized to specific connections or subnetworks. The network-based statistic (NBS)^18^ identified a single subnetwork of connections with significantly increased functional connectivity in the fasted group (p = 0.01, family-wise error corrected). The subnetwork comprised 9 connections that spanned a total of 6 ROIs and included the left and right hippocampus as well as several regions comprising the left temporal cortex (Fig. 4a). Functional connectivity values for two of the connections comprising this subnetwork are shown as boxplots in Fig. 4b,c (Data are expressed as mean ± SEM.). The increase in functional connectivity for each connection comprising this subnetwork exceeded a Cohen’s d of 0.7 (medium-to-large effect size). This result suggests that the global increase in functional connectivity is principally attributable to the hippocampus, retrosplenial cortex, and regions in the temporal cortex. We did not identify any subnetworks with significant decreases in functional connectivity in the fasted group (p > 0.05).

### Decreased ALFF in the ventromedial hypothalamus

**Figure 5:**
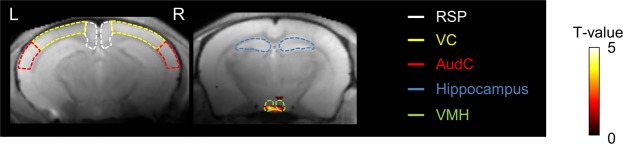
Pixel-by-pixel analysis of significant increase of ALFF in non-fasted group compared with that in fasted group (p < 0.001, uncorrected). Color bar, t-value. AudC, auditory cortex; RSP, retrosplenial cortex; LD, laterodorsal thalamus; VC, visual cortex; VMH, ventromedial hypothalamus.

ALFF was found to significantly differ between the non-fasted and fasted groups (Fig. 5). Voxel-wise comparison of ALFF between the two groups identified a significant decrease in ALFF in the ventromedial hypothalamus in the fasted group, but there were no significant changes in the retrosplenial cortex, visual cortex, auditory cortex and the hippocampus (Fig. 5).

### Correlation of the neuronal oscillation

**Figure 6:**
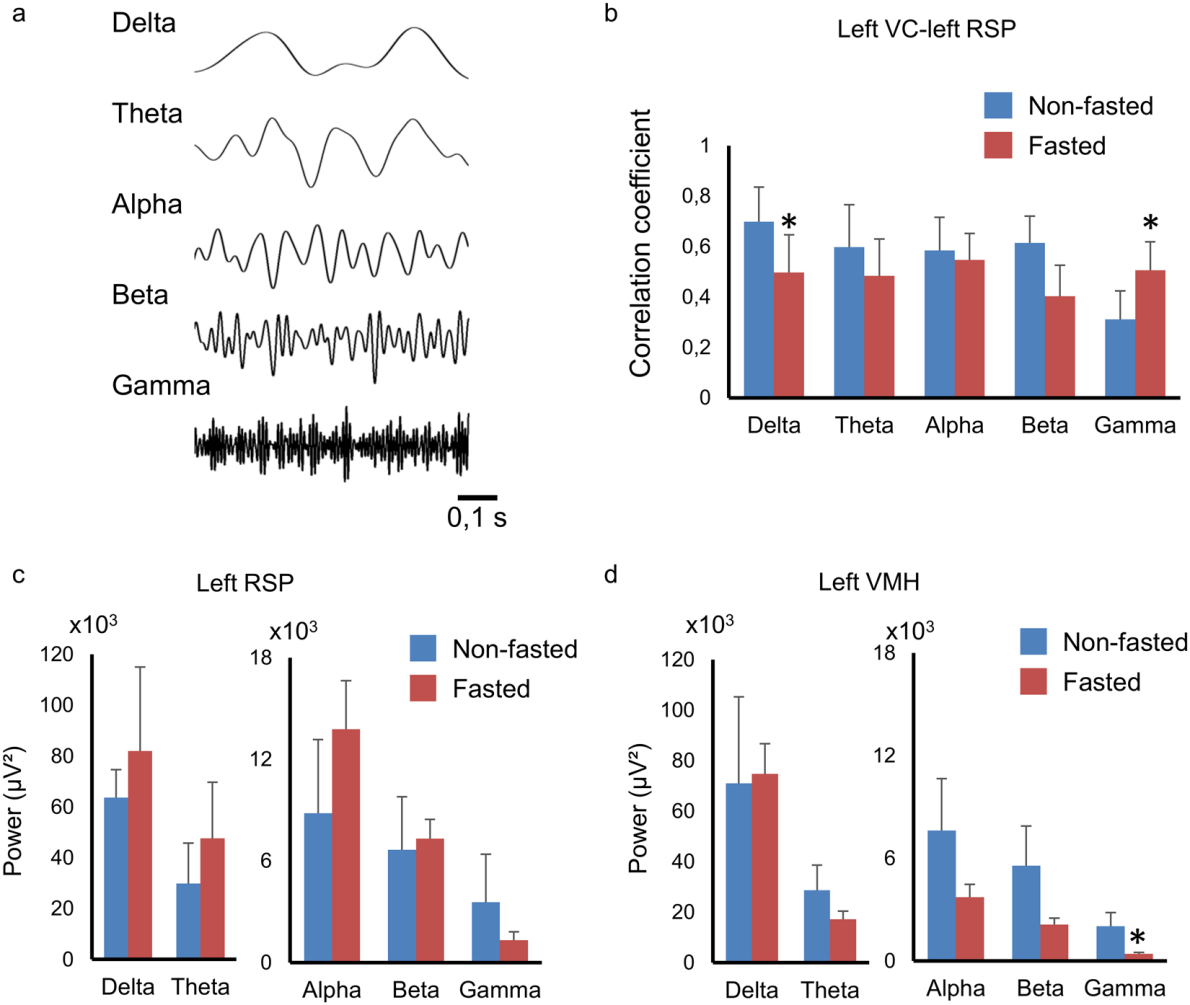
(**a**) Representative waveform of filtered recordings of alpha, beta and gamma oscillations. (**b**) Correlation coefficients of delta, theta, alpha, beta and gamma oscillations between left visual cortex and left retrosplenial cortex (left VC-left RSP) in fasted (n = 6) and non-fasted groups (n = 6). Power of each oscillation (**c**) in left retrosplenial cortex (left RSP) and (**d**) in left ventromedial hypothalamus (left VMH) in fasted (n = 6) and non-fasted groups (n = 6). The data are reported as mean ± SEM. *p < 0.05 by t-test in each frequency band.

We next sought to test whether the fMRI-measured increases in functional connectivity induced by fasting could be validated using intracerebral LFP. We specifically focused on the synchrony of band limited power (BLP) of LFP delta, theta, alpha, beta and gamma oscillations between the retrosplenial and visual cortices, regions between which functional connectivity was significantly increased in the fasted group. Fig. 6a shows representative LFP oscillations for each frequency band. In agreement with the fMRI data, synchrony in gamma oscillations between left retrosplenial and left visual cortices was significantly increased in the fasted group compared to the non-fasted group, while synchronicity in delta oscillations was decreased (Fig. 6b, p < 0.05). This suggests that increases in functional connectivity induced by fasting are most likely explained by a coupling of gamma oscillations. Synchrony in alpha and beta oscillations did not significantly differ between groups (Fig. 6b). In further analyses, the power of each frequency band was investigated in left retrosplenial cortex and left ventromedial hypothalamus (Fig. 6c,d). The power of gamma oscillations in left ventromedial hypothalamus was significantly decreased in the fasted group compared to the non-fasted group but there was no significant change in delta, theta, alpha and beta oscillations (Fig. 6d). No significant change was observed in left retrosplenial cortex (Fig. 6c). There was no significant difference in the total LFP power in the ventromedial hypothalamus (data not shown).

### Network modularity

**Figure 7:**
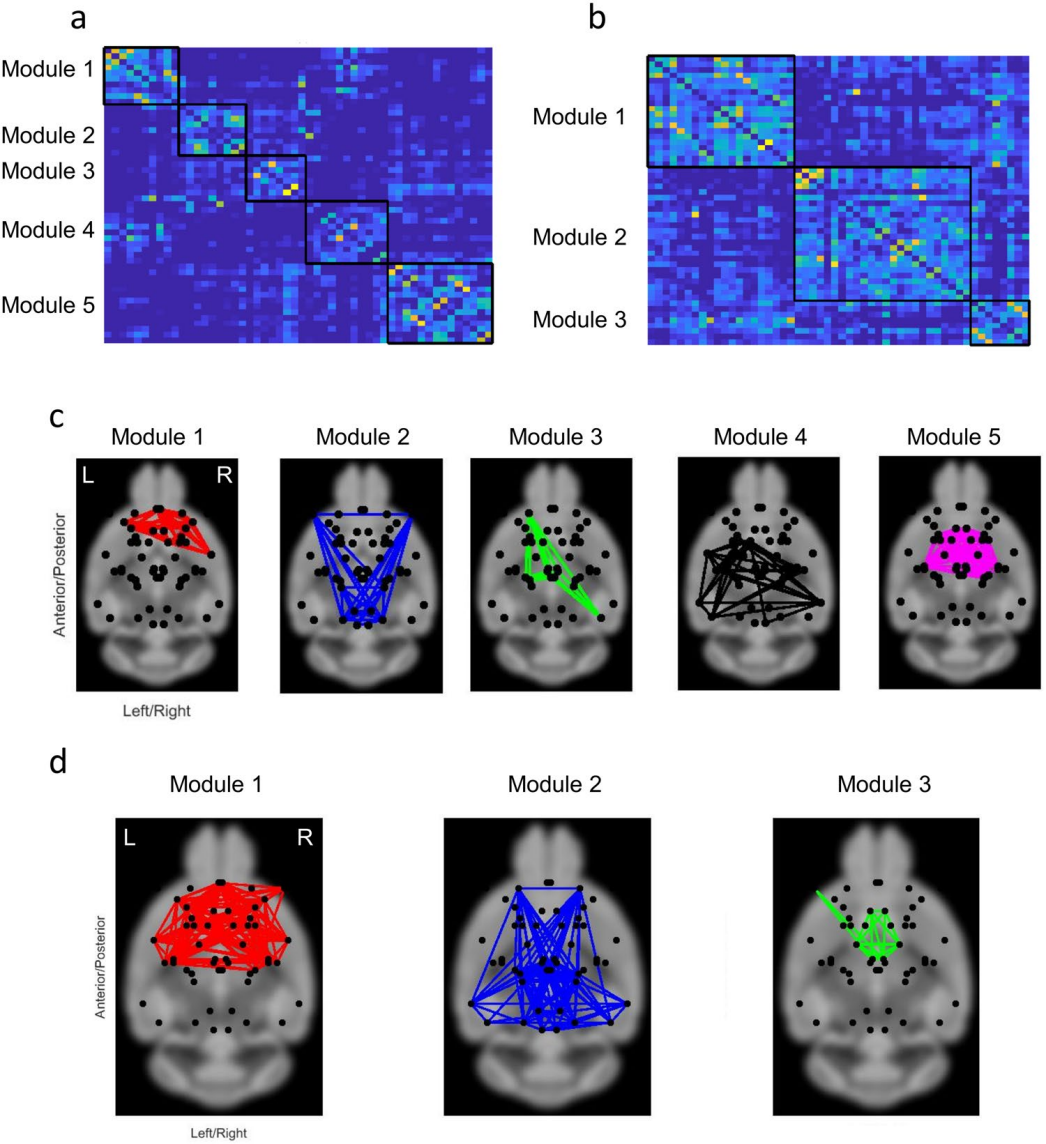
(**a**) Association matrix for calculating modules. The modules in (**b**) non-fasted and (**c**) fasted groups. Background image from http://imaging.org.au/AMBMC/Model.

Finally, we sought to characterize the modular organization of functional brain networks in the fasted and non-fasted groups. The Louvain algorithm was used to decompose the functional brain network of each individual mouse into modules and an iterative consensus clustering approach was used to derive a module structure that was representative of each group. The functional brain networks of both groups showed modular organization (non-fasted: Q = 0.60 ± 0.08; fasted: Q = 0.57 ± 0.07), but Q-scores did not significantly differ between the two groups (p > 0.05). However, the functional brain networks of the non-fasted group comprised 5 modules, whereas the fasted group comprised only 3 modules (Fig. 7). This suggests that fasting is associated with a loss of normal segregation of functional brain networks.

## Discussion

Physiological states such as hunger and satiety are known to influence mood, cognitive performance and feeding behavior. Here, we used high-field functional MRI to demonstrate that fasting in mice is associated with hyper-connectivity, most prominently involving connections of the hippocampus, retrosplenial cortex, visual cortex and the auditory cortex. We found that ALFF measurements, which relate to the power of the electrical current oscillations caused by excitatory neuronal activity^19,20^, were decreased in the glucose-sensitive ventromedial hypothalamus in the fasted group, while no changes were detected in the retrosplenial cortex and visual/auditory cortex. Neuronal recordings suggested that these alterations were due to reduced neuronal activity and increased interregional synchrony in gamma oscillations. Our study provides new insight into the neuronal mechanisms of functional connectivity changes induced by fasting and suggests that altered neurovascular coupling due to reduced blood glucose levels is unlikely to be the principal mechanism underlying these changes. Our study also points to the importance of maintaining a stable energy state for animal studies, given that an animal’s satiety can profoundly impact functional connectivity.

The advantage of resting state fMRI is that we can investigate a putative measure of neural synchronicity across the entire cortex non-invasively. We emphasize that while immediate-early genes (IEG) and C-Fos expression are powerful techniques that can index neuronal activity^21^, they do not provide insight into functional connectivity, which specifically characterizes synchrony between spatially separated regions.

### Blood glucose levels and brain function

It is important to discuss the effect of blood glucose levels on BOLD signal changes because glucose metabolism is essential to neuronal activity. It is well known that the BOLD response is highly sensitive to the neurovascular circumstances and to vasoactive drugs and metabolites^22–26^. However, previous studies have suggested that changes in acute blood glucose level do not affect BOLD responses^27^. We found that blood glucose levels were approximately 25% lower in the fasted mice. This is consistent with previous studies, which report a decrease of blood glucose levels in the range of 16–26% in fasting conditions (9–29 h)^4^.

The fluctuation of blood glucose levels induces neuronal activity in the hypothalamic neurons^28^. The ventromedial regions of the hypothalamus do indeed comprise specialized cells — “glucose-sensing” neurons — that respond to changes in extracellular glucose concentration by varying their firing rate. Blood glucose increase by glucose intake induces neuronal activity in the hypothalamic nuclei including the ventromedial hypothalamus^29–32^. The fasted group showed significant decrease of both blood glucose levels and ALFF in the ventromedial hypothalamus (Figs 1 and 4). This result is in agreement with previous studies^33^. Remarkably, functional connectivity of the hypothalamus network did not change. This suggests that the glucose-sensitive neuronal activity in the ventromedial hypothalamus did not interfere with functional connectivity.

### Mechanism of altered functional connectivity: altered neurovascular coupling or neuronal activity?

Blood glucose levels both in the non-fasted and fasted groups were higher than those of awake rodents^34^. This is a side-effect of anesthetization with isoflurane, which increases blood glucose levels as well as the vasodilation^35^. However, neurovascular coupling is not fully disrupted under a low dose of isoflurane^36–38^. Increased blood glucose levels were within a normal range, compared with acute hyperglycemia, which potentially changes cerebral blood flow^39,40^. It is important to remark that administration of low doses of anesthesia, including isoflurane, can potentially results in alterations of the strength of functional connectivity compared with awaked state^41^.

Alterations in metabolism and blood glucose levels induced by fasting can potentially influence neurovascular coupling and thus affect BOLD activity and functional connectivity. However, we found that synchrony in gamma oscillations derived from LFP were significantly increased and synchrony in delta oscillations were significantly decreased in the fasted group between a pair of regions that also showed increased functional connectivity derived from fMRI (i.e. left retrosplenial and left visual cortices). Given that LFP is not directly impacted by neurovascular effects, this suggests that the increases in functional connectivity induced by fasting most likely did not owe to altered neurovascular coupling. We also found that the power of gamma oscillations was decreased in the ventromedial hypothalamus in the fasted group, compared to the non-fasted group. Previous studies report the impact of gamma oscillations in the lateral hypothalamus on food seeking behavior^42^. The theta band activity is also associated with locomotor activity in lateral hypothalamus^43^. As mice were anesthetized in this study, we did not observed food seeking behavior and synchronicity of gamma oscillation in the lateral hypothalamus. Our findings are consistent with previous rodent and primate studies that suggest a link between gamma oscillations and BOLD-derived functional connectivity^19,44^, and point to a neuronal origin, rather than neurovascular effects, of the observed increases in functional connectivity associated with fasting. Additionally, James *et al*. showed that negative delta oscillation-BOLD correlation and positive gamma oscillation-BOLD correlation in resting state fMRI in rats^45^. The high and low LFP–BLP envelopes are also significantly correlated with spontaneous BOLD activity recorded from positively and negatively connected prefrontal network regions in the macaque monkeys^46^. Together with our data, this suggests that low frequency (delta) oscillations as well as gamma oscillation could contribute to the correlation of the BOLD fluctuations.

### Connectivity between the visual/auditory cortex, retrosplenial cortex and hippocampus

We found that fasting results in increased resting-state functional connectivity in the hippocampus-cortex pathway which includes the retrosplenial, visual and auditory cortices. Two-photon calcium imaging shows that neurons in the mouse postrhinal association cortex, which likely serves as a relay of visual object information to the hippocampus, exhibits biases towards food-associated cues in hunger states^47^. Moreover, the retrosplenial cortex can support cognitive decisions that are the consequence of visual or auditory inputs^48^. This suggests that the observed increases in functional connectivity among the retrosplenial cortex, hippocampus and visual/auditory cortex could influence food seeking behavior. Human fMRI studies of hunger report that fasting induces increases in functional connectivity between the superior frontal gyrus and insular cortex^16^. This change was not found in our mouse model, which may be due to distinct feeding cycles^49^. Mice consume most of their food in frequent, small meals throughout the night, whereas humans tend to consume three or four meals during the day. Therefore, overnight fasting in humans is not necessarily equivalent to fasting in mice. Further study is needed to understand the effects of daytime fasting in mice. Interestingly, the increase of connectivity in the left hemisphere was observed in the fasting state. While the study of functional connectivity laterality in the rodent brain is more limited than human study^50^, some studies discuss asymmetries in cerebral cortical networks in non-human species and there is evidence of laterality of non-human species, including mice and chicks^51^. Previous studies suggest that ultrasound communication in the mouse is a function that is lateralized to the left hemisphere^52^. The volume of the right hemisphere is larger than that of the left hemisphere in mice and rats^53^. However, as the mechanism of the laterality in mice is not clear, further study is required. This study provides evidence that fasting is associated with an asymmetrical change in functional connectivity.

Interestingly, the null hypothesis of equality in mean ALFF between the fasted and non-fasted groups was not rejected for regions of the hippocampus, retrosplenial, auditory and visual cortices. Given that the low frequency fluctuations of BOLD measured by ALFF are thought to be coupled to the underlying fluctuations of excitatory neuronal activity^19^ and reflect the integration and summation of the input and local processing of neural activity like LFP^54^, we suggest that the increases in functional connectivity found in the fasted group are not exclusively due to glucose-dependent mediation of neuronal activity.

### Network modularity

Functional brain networks showed evidence of modular organization in both the fasted and non-fasted group. However, functional networks in the fasted group comprised fewer modules, suggesting a loss of network segregation. Remarkably, the number of nodes in each community of the cingulate cortex, hippocampus, retrosplenial, visual and auditory cortices (module 1 and 2 in non-fasted group and module 2 in fasted group), was increased in fasted mice. Additionally, the community including the hypothalamus and thalamus was found in the fasted and non-fasted groups, although the size of the community decreased in the fasted group (module 5 in non-fasted group and module 3 in fasted group).

### Limitations

In the present study, blood glucose levels were not measured in all mice following MRI scanning. Therefore, it was not possible to investigate potential associations between the strength of functional connectivity and blood glucose levels.

Although several previous functional connectivity studies use sample sizes comparable to this study (8 fasted mice vs 11 non-fasted mice), caution is needed when interpreting findings from small sample sizes because effects may be owing to individual differences^7,55,56^, rather than fasting. Our findings therefore require validation in a larger sample.

In the present study, artificial regulation of the respiration by means of ventilation was not used. Although previous studies showed the functional connectivity without ventilation^8,12,55^, it is possible that the scanning in the absence of intubation in an anesthetized state could potentially lead to variation in brain state and physiology.

Another limitation is the relative coarseness of the cortical and subcortical parcellations used. For example, the hippocampus was represented as a single ROI, without characterization of its inherent sub-regions (e.g., CA1, CA3 and dentate gyrus). However, for whole-brain analyses of functional connectivity, parcellation atlases of the coarseness used in this study are typical and using finer parcellations can reduce statistical power due to inherent increases in the number of multiple comparisons^14,57,58^.

## Conclusion

We acquired high-field fMRI in groups of fasted and non-fasted mice to investigate the impact of fasting on functional connectivity and the organization of functional brain networks. We found that fasting induced widespread increases in functional connectivity, with the most significant increases circumscribed to the hippocampus, the visual/auditory cortex and the retrosplenial cortex. Analysis of electrophysiological data suggested that these increases were most likely neuronal in origin and not entirely related to neurovascular coupling. We also found that fasting was associated with decreased functional network segregation, evidenced by fewer network modules found in the fasted group, and decreased neuronal activity in the ventromedial hypothalamus. The functional connectivity changes associated with fasting may be linked to altered feeding patterns, cognitive performance and learning.

## Materials and Methods

### Animals

Thirty-one male SWISS mice (25–35 g, Janvier, Janvier Labs, Saint Berthevin, France) were allocated randomly into one of four groups: i) non-fasted (n = 11) and fasted (n = 8) groups for the fMRI study; and, ii) non-fasted (n = 6) and fasted (n = 6) for the electrophysiological study. Following the fMRI study, blood glucose levels were measured in several mice (non-fasted, n = 4; fasted, n = 4). The animals were housed in wire-mesh cages under controlled temperature (23 °C), humidity (30%) and light (7:00–19:00) conditions with free access to water and food. Experiments were performed more than 1 week after arrival of the mice for adopting the housing circumstance. All *in vivo* animal procedures in the present study were approved by Comité d’Ethique en Expérimentation Animale, Commissariat à l’Energie Atomique et aux Énergies Alternatives, Direction des Sciences du Vivant (Fontenay-aux-Roses, France) and by the Ministère de l’Education Nationale de l’Enseignement Supérieur de la Recherche (France). All methods in this study were performed in accordance with the relevant guidelines and regulations. Mice in the fasted group were denied access to food for 12 h before the start of scanning (from the night).

### Blood glucose levels

Blood glucose level was measured from tail-tip blood using a blood glucose meter (OneTouch Verio, LifeScan. Inc., CA) under 0.8–1.0% isoflurane anesthesia.

### MRI acquisition

All MRI experiments were performed with a Bruker 11.7 T MRI system with cryoprobe (Bruker BioSpin, Ettlingen, Germany). Each mouse was lightly anesthetized with isoflurane (1.5% for induction and 0.8–1.0% for maintenance) in air containing 30% O_2_. The head was fixed using ear-bars and a teeth-bar. Respiration rate and body temperature were monitored (model 1025; SA Instruments, NY, USA). Respiration rates were constant throughout fMRI scanning, ranging between 80–120 beat per min for all mice. The null hypothesis of equality in mean respiration rates between the groups of fasted and non-fasted mice was not rejected with a two-sample *t*-test (p = 0.85). Body temperature was maintained at 37 °C using circulating hot water. The blood oxygenation level dependent (BOLD) fMRI data were obtained using a T2*-weighted multi-slice echo-planar imaging (EPI) sequence with the following parameters: repetition time = 2 s, echo time = 12 ms, field of view = 15 mm × 9.2 mm, acquisition matrix = 150 × 92 (0.1 × 0.1 mm²/pixel), slice thickness = 0.5 mm and number of slices = 15. A total of 180 volumes were acquired for each mouse, equivalent to a 6 min acquisition interval. An acquisition of this duration is adequate to investigate functional connectivity in mice^55,56^. Structural images with the same field of view and resolution as the fMRI were obtained with a multi-slice rapid acquisition with relaxation enhancement (RARE) sequence using the following parameters: time of repetition = 2,500 ms, effective echo time = 13 ms, RARE factor = 4, and 4 averages.

### Data preprocessing

The resting state fMRI data analysis toolkit (REST1.8, Lab of Cognitive Neuroscience and Learning, Beijing Normal University, China) and SPM8 software (Wellcome Trust Centre for Neuroimaging, UK) were used to analyze the fMRI data. In brief, preprocessing steps included slice timing, realignment, co-registration to structural images and spatial normalization of functional data. Before preprocessing, template images co-registered to the Paxinos and Watson mouse brain atlas were obtained (Paxinos G and C Watson 1998)^59^. The functional and structural images were normalized to these template images. Mean signals in the ventricles and white matter, and six motion parameters of an object (the translational and rotational motion) from SPM8 were regressed out from the time-series of each voxel to reduce contributions from physiological noise. The preprocessed fMRI data were then detrended and slow periodic fluctuations were extracted using a bandpass filter (0.01–0.1 Hz). The residuals resulting from this regression were then smoothed with a Gaussian filter (0.2 × 0.2 × 0.5 mm^3^). The smoothed residuals were used for all subsequent analyses in ALFF and functional connectivity. These steps were performed separately for each mouse.

### Functional connectivity

A total of 52 regions-of-interest (ROIs, 26 per hemisphere) were manually delineated based on the template images, with reference to the mouse brain atlas as described above, using MRIcron (http://www.mccauslandcenter.sc.edu/mricro/mricron/). Table 1 lists all ROIs and each ROI has 80–2000 voxels. A ROI-averaged time-series was generated for each ROI by averaging the fMRI data (smoothed residuals) across all voxels encapsulated by the ROI. Functional connectivity was measured between each of the (52 × 51)/2 = 1326 unique pairs of nodes, resulting in a 52 × 52 functional connectivity matrix for each mouse.

**Table 1:** Regions-of-interest (ROIs) and abbreviations.

Abbreviation	ROI
S1_ll	Primary somatosensory cortex lower limb
S1_ul	Primary somatosensory cortex upper limb
S1_BF	Primary somatosensory cortex barrel field
MC	Motor cortex
IC	Insular cortex
aCC	Anterior cingulate cortex
RSP	Retrosplenial cortex
AudC	Auditory cortex
VC	Visual cortex
Hippocampus	Hippocampus
BLA	Basolateral amygdala
CeA	Central amygdala
CM	Central medial nucleus of the thalamus
MD	Mediodorsal nucleus of the thalamus
VPL	Ventral posterolateral nucleus of the thalamus
VPM	Ventral posteromedial nucleus of the thalamus
LH	Lateral hypothalamus
VMH	Ventromedial hypothalamus
MPA	Medial preoptic area
dCPu	Dorsal caudate putamen
GPi	Globus pallidus, internal segment
GPe	Globus pallidus, external segment
VP	Ventral pallidum
Acb	Nucleus accumbens
VTA	Ventral tegmental area
SN	Substantia nigra

### Amplitude of low frequency fluctuations

The motivation for investigating ALFF was to provide insight into whether any changes in functional connectivity associated with fasting were related to changes in the amplitude of neural activity. ALFF is defined as the total power within the frequency range between 0.01 and 0.1 Hz for each voxel to generate an ALFF map for each mouse. The ALFF value in each voxel was divided by the averaged ALFF value in the whole brain. The null hypothesis of equality in mean ALFF between the fasted and non-fasted group was tested independently for each voxel using a two-sample *t*-test using SPM (p < 0.001, uncorrected).

### Network-based statistic

The NBS^18^ was used to test the null hypothesis of equality in functional connectivity between the fasted and non-fasted groups. The NBS was performed as follows: for each of the 52 × 51/2 = 1326 unique pairs of ROIs (functional connections), a two-sample *t*-test was used to independently test whether group-averaged functional connectivity differed between the two groups. This yielded a 52 × 52 matrix of *t*-statistic values. Any connection with a *t*-statistic below 3 was excluded from further testing, which ensured that the null hypothesis could only be rejected for between-group differences with a Cohen’s *d* exceeding 0.7 (minimum meaningful effect size). This threshold was chosen to ensure conclusions were only drawn on the basis of medium-to-large effect sizes. Subnetworks were then identified in the set of supra-threshold connections. A subnetwork, or connected component, is a set of ROIs for which a path can be found between any pair of ROIs in the set of supra-threshold connections. The size of each subnetwork was measured in terms of the number of connections it comprised. Permutation testing was used to compute a family-wise error corrected p-value for each subnetwork. Specifically, 5000 permutations were generated in which group labels were randomly permuted and the size of the largest subnetwork was recorded for each permutation. Storing the size of largest subnetwork for each permutation resulted in a null distribution that ensured control of the familywise error rate. A familywise error corrected p-value for each subnetwork in the actual data was computed as the proportion of permutations for which a subnetwork of larger or equal size was found. By seeking to reject the null hypothesis at the level of groups of connections (i.e. subnetworks), rather than individual connections, the NBS can substantially improve statistical power. Between-group differences are seldom confined to individual connections, and thus the NBS seeks to identify effects that are spread across groups of related connections that form subnetworks. Subnetworks deemed to be significant with permutation testing (p < 0.05, familywise error corrected) were visualized to enable anatomical localization. These steps were performed with the NBS Connectome software (www.nitrc.org/projects/nbs/).

### Modularity

Functional brain networks are organized into modules^60^. A module refers to a community of brain regions (ROIs) that are more strongly (or more densely) interconnected than expected under an appropriate null model. Modules are thought to enable functional specialization and facilitate the emergence of complex neural dynamics. We aimed to characterize the modular structure of functional brain networks in the fasted and non-fasted groups. We employed the Louvain algorithm^61^ to decompose the 52 × 52 functional connectivity matrices into modules for each individual mouse and an iterative consensus clustering^62^ to characterize group-level modular structure. In brief, the Louvain algorithm was used to decompose the functional connectivity matrix of each individual mouse into modules. For each group, a 52 × 52 co-classification matrix was then populated, where element (*i*, *j*) stored the proportion of mice for which ROI *i* and ROI *j* resided in the same module. The co-classification matrix was thresholded so that any elements with a value below 0.4 were set to zero. The threshold of 0.4 has previously been found to provide good performance^62^. The Louvain algorithm was then applied to the thresholded co-classification matrix to determine a single modular decomposition that was representative of the group. This was repeated for the fasted and non-fasted groups. To alleviate modularity degeneracy^63^, rather than computing only a single modular decomposition for each individual mouse, we computed 100 decompositions per mouse using the Louvain algorithm. A consensus across these 100 decompositions was then computed. Therefore, iterative consensus clustering was used to identify a consensus modular structure: i) for each of the 100 decompositions generated for each individual mouse; and then, ii) for each group. The extent of modular structure was quantified with the Q-score^64^ and the resolution parameter for the Louvain algorithm was set to 1. Negative correlation coefficients were set to 0 in the functional connectivity matrices due to their ambiguous interpretation^65^. These modularity analyses were undertaken with Brain Connectivity Toolbox (BCT; https://sites.google.com/site/bctnet/).

### LFP recording

Electrophysiological recordings were performed separately outside the MRI bore. The animals, first anesthetized with 1.5% isoflurane, were placed in a stereotaxic frame (David Kopf instrument, CA). Body temperature was maintained at 37 °C using a heating pad (DC temperature controller; FHC Inc., Bowdoin, ME, USA). The skull was exposed and multiple holes (1 mm diameter) were made through the skull with a dental drill for insertion of micro-electrodes. The multiple tungsten microelectrodes (<1.0 MΩ, with 1 μm tip and 0.127-mm shaft diameter, Alpha Omega Engineering, Nazareth, Israel) was positioned at left visual cortex (AP −2.9 mm, ML −2.5 mm, DV −0.8 mm from the bregma), left retrosplenial cortex (AP −2.3 mm, ML −0.3 mm, DV −0.8 mm from the bregma) and left ventromedial hypothalamus (AP −1.6 mm, ML −0.3 mm, DV −5.5 mm from the bregma). After surgery, isoflurane concentration was changed to 0.8–1.0%, which is the same concentration as the fMRI experiment. Electrodes were connected to a differential AC amplifier Model 1700 (AM systems, Sequim, WA, USA), via a Model 1700 head stage (AM systems, Sequim, WA, USA). LFPs were continuously recorded for 6 min. LFP signals in three regions were simultaneously acquired at 10 kHz sampling rate using dedicated data acquisition software (Power Lab, AD Instruments, Dunedin, New Zealand). The reference electrode was positioned on the scalp.

### LFP analysis

From LFP signal, five BLP time series were calculated: delta (1–4 Hz), theta (4–8 Hz), alpha (8–12 Hz), beta (18–30 Hz) and gamma (60–100 Hz) using PowerLab (AD Instruments, Dunedin, New Zealand)^46,66^. The pearson correlation coefficients of the time-series of each BLP (delta, theta, alpha, beta or gamma frequency) were then computed between the pair of recorded regions (visual cortex and retrosplenial cortex). The mean power of each frequency band in the retrosplenial cortex and ventromedial hypothalamus was calculated for each frequency range.
