# Author Correction: The impact of fasting on resting state brain networks in mice

**DOI:** 10.1038/s41598-020-61221-w

**Published:** 2020-03-03

**Authors:** Tomokazu Tsurugizawa, Boucif Djemai, Andrew Zalesky

**Affiliations:** 1 NeuroSpin, Commissariat à l’Energie Atomique et aux Energies Alternatives, CEA Saclay, Gif-sur-Yvette, 91191 France; 2 Melbourne Neuropsychiatry Centre and Department of Biomedical Engineering, The University of Melbourne, Victoria, 3010 Australia

Correction to: *Scientific Reports* 10.1038/s41598-019-39851-6, published online 27 February 2019

This Article contains errors in the legend of Figure 7.

“a) Association matrix for calculating modules. The modules in (b) non-fasted and (c) fasted groups. Background image from http://imaging.org.au/AMBMC/Model.”

should read:

“Association matrix for calculating modules in (a) non-fasted and (b) fasted groups. The modules in (c) non-fasted and (d) fasted groups. Background image from http://imaging.org.au/AMBMC/Model.”

